# Dynamics of spinal microglia repopulation following an acute depletion

**DOI:** 10.1038/srep22839

**Published:** 2016-03-10

**Authors:** Yao Yao, Stefania Echeverry, Xiang Qun Shi, Mu Yang, Qiu Zi Yang, Guan Yun Frances Wang, Julien Chambon, Yi Chen Wu, Kai Yuan Fu, Yves De Koninck, Ji Zhang

**Affiliations:** 1 The Alan Edwards Centre for Research on Pain, McGill University, Montreal, QC, Canada; 2 Department of Neurology & Neurosurgery, McGill University, Montreal, QC, Canada; 3 Faculty of Dentistry, McGill University, Montreal, QC, Canada; 4 Center for TMD & Orofacial Pain, Peking University School & Hospital of Stomatology, Beijing, China; 5 Institut universitaire en santé mentale de Québec, QC G1J 2G3, Canada; 6 Department of Psychiatry & Neuroscience, Université Laval, Québec, G1V 0A6, Canada

## Abstract

Our understanding on the function of microglia has been revolutionized in the recent 20 years. However, the process of maintaining microglia homeostasis has not been fully understood. In this study, we dissected the features of spinal microglia repopulation following an acute partial depletion. By injecting intrathecally Mac-1-saporin, a microglia selective immunotoxin, we ablated 50% microglia in the spinal cord of naive mice. Spinal microglia repopulated rapidly and local homeostasis was re-established within 14 days post-depletion. Mac-1-saporin treatment resulted in microglia cell proliferation and circulating monocyte infiltration. The latter is indeed part of an acute, transient inflammatory reaction that follows cell depletion, and was characterized by an increase in the expression of inflammatory molecules and by the breakdown of the blood spinal cord barrier. During this period, microglia formed cell clusters and exhibited a M1-like phenotype. MCP-1/CCR2 signaling was essential in promoting this depletion associated spinal inflammatory reaction. Interestingly, ruling out MCP-1-mediated secondary inflammation, including blocking recruitment of monocyte-derived microglia, did not affect depletion-triggered microglia repopulation. Our results also demonstrated that newly generated microglia kept their responsiveness to peripheral nerve injury and their contribution to injury-associated neuropathic pain was not significantly altered.

Although neurons in the central nervous system (CNS) have limited capacity for regeneration, glial cells exhibit remarkable self-renewal potential. Aroused from yolk sac progenitors that populate the CNS during embryogenesis, microglia in adulthood has been well recognized for their capability in preserving local homeostasis. Failure to keep up microglia in their normal physiological states leads to alteration in the stability of CNS micro-environment, as microglia are not only overseers of pathological disturbances^1^,^2^ they also have physiological roles in normal CNS function^3^,^4^. However, the question of how microglia strive to maintain the integrity of the cell population is intriguing and unresolved, it has drawn much attention in recent research of microglia cell biology. Several research groups have investigated microglia repopulation after depletion in the brain parenchyma using genetic and/or pharmacological approaches. The main findings have identified the CNS resident microglia as the cell population responsible for re-establishing the CNS microglia compartment. Elmore *et al.*^5^ reported that following depletion by blocking colony-stimulating factor1 receptor (CSF1R) signaling, microglia can repopulate solely through proliferation of nestin-positive, resident cells which then differentiate into microglia. The notion that microglia repopulation relies fully on CNS resident cells is further supported by the group of Bruttger^6^ where Cx3cr1^CreER^:iDTR system has been used to ablate microglia cells. The participation of bone marrow-derived cells in the regeneration process has also been reported^7^, but was considered to occur only in pre-conditioned environments^6^. Whereas cells contributing to microglia recovery have been identified, the details along with the repopulation process have not been fully analyzed.

As the microglia population shows remarkable anatomical, morphological and functional diversity in each area of the CNS^8^,^9^, different functional organizations (e.g., brain, spinal cord and retina) harboring microglia have distinct activation thresholds that are primed to respond differently to insults. Although microglia responsiveness to local depletion and their capability to reset to steady status have been explored in the brain, very few if any, of such studies with regard to the spinal cord can be found in the literature. The spinal cord is the first relay site in the transmission of nociceptive information from the periphery to the brain. Spinal microglia become activated following an injury to peripheral nerve^10^,^11^ and are important players in the pathogenesis of neuropathic pain^12^,^13^. In addition, spinal microglia have functional contribution to secondary damage after spinal cord injury (SCI)^14^. Microglia have been considered as potential targets of immune modulatory therapies for chronic pain and SCI^15^. It is thus of great value to understand the details of spinal microglia behavior in response to local spinal depletion.

In this study, we aim to address the following questions: Can spinal microglia repopulate in a timely manner following local cell depletion? Does spinal microglia repopulation depend on resident or monocyte-derived microglia or both? And could newly generated microglia preserve the same functional characteristics as their original counterparts? Special attention has been paid to the events occurring during the repopulation process. To this end, we depleted spinal microglia by intrathecal injection of a microglia selective immunotoxin, Mac-1-saporin, which resulted in a rapid, re-establishment of microglia homeostasis within 14 days. Our results support the concept that microglia repopulation, whether in the brain or in the spinal cord, is the consequence of *onsite* resident microglia proliferation. Although circulating monocyte infiltration was observed shortly after the depletion, this appears to be part of cell death-triggered, MCP-1/CCR2 signaling dependent inflammation, which is, interestingly, not required for the microglia repopulation process. Newly generated microglia are fully functional. They are able to respond to peripheral nerve injury and contribute to the development of neuropathic pain.

## Results

### Spinal microglia repopulation occurs shortly after an acute depletion

**Figure 1:**
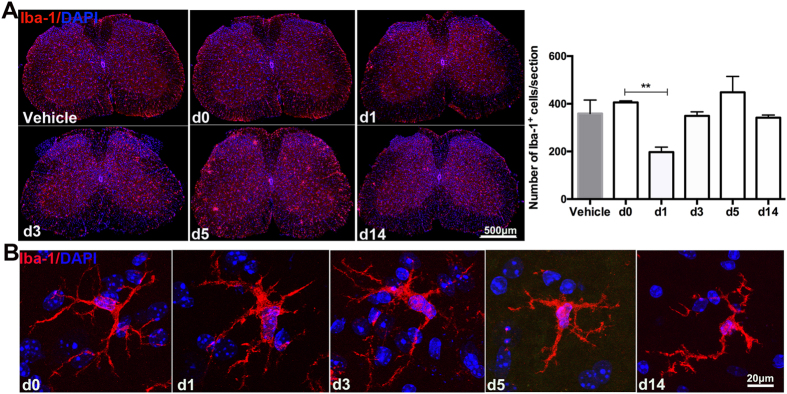
Spinal microglia cell density and morphology changes following an acute cell depletion. (**A**) Representative examples of IHC analysis depicted that the number of Iba-1^+^ microglia reduced to about 50% 1 day after one single intrathecal injection of Mac-1-saporin, but it quickly recovered to the baseline level 3 days post-depletion. At day 5, there was a burst of Iba-1^+^ cell clusters formed within the parenchyma. Iba-1^+^ cell density was stabilized at 2 weeks post-depletion. Quantification analysis on the number of microglia was performed on the entire section of the lumbar spinal cords, 5 sections/mouse, 3–7 animals/group. *p < 0.05. (**B**) Representative examples of IHC images demonstrated depletion-triggered microglia morphology changes at different time points. At early phase (day 1–day 5) post-depletion, Iba-1^+^ cell exhibited hypertrophic morphology, with enlarged cell bodies, thickened and shortened processes; The most prominent changes were found at day 1 post-depletion, while at day 14, microglia displayed ramified shapes not fully differentiated into their original status (day 0) yet.

To understand the dynamic process of spinal microglia repopulation, we made use of a microglia selective immunotoxin, Mac-1-saporin, to first deplete locally microglia within lumbar spinal cord. One day after intrathecal injection of Mac-1-saporin (7 μl, 1.6 μg/μl) at L4-L5 level, the number of Iba-1^+^ microglia in the lumbar spinal cord reduced to 50% of those mice without depletion (Fig. 1A). Microglia repopulation occurred rapidly following the acute partial depletion (Fig. 1A). At day 3 post-Mac-1-saporin injection, the number of Iba-1^+^ cells reached already the same level before depletion. The total number of microglia was stabilized at day 14. Clusters grouped by ≥3 Iba-1^+^ cells were found disseminated within the spinal parenchyma, mainly at the early phase, day 3–5 post-Mac-1-saporin injection. Very few Iba-1^+^ clusters were detected at day 14. In addition, following depletion, microglia displayed hypertrophic morphology with enlarged cell bodies, thickened and shortened processes. While the most striking morphological changes appeared at the early depletion-repopulation period (day 1–5), microglia at 14 days post-depletion exhibited essentially a ramified shape, although not yet differentiated fully into their original states before depletion (Fig. 1B).

### Microglia depletion triggers cell proliferation and bone marrow derived-cell infiltration into the spinal cord parenchyma

**Figure 2:**
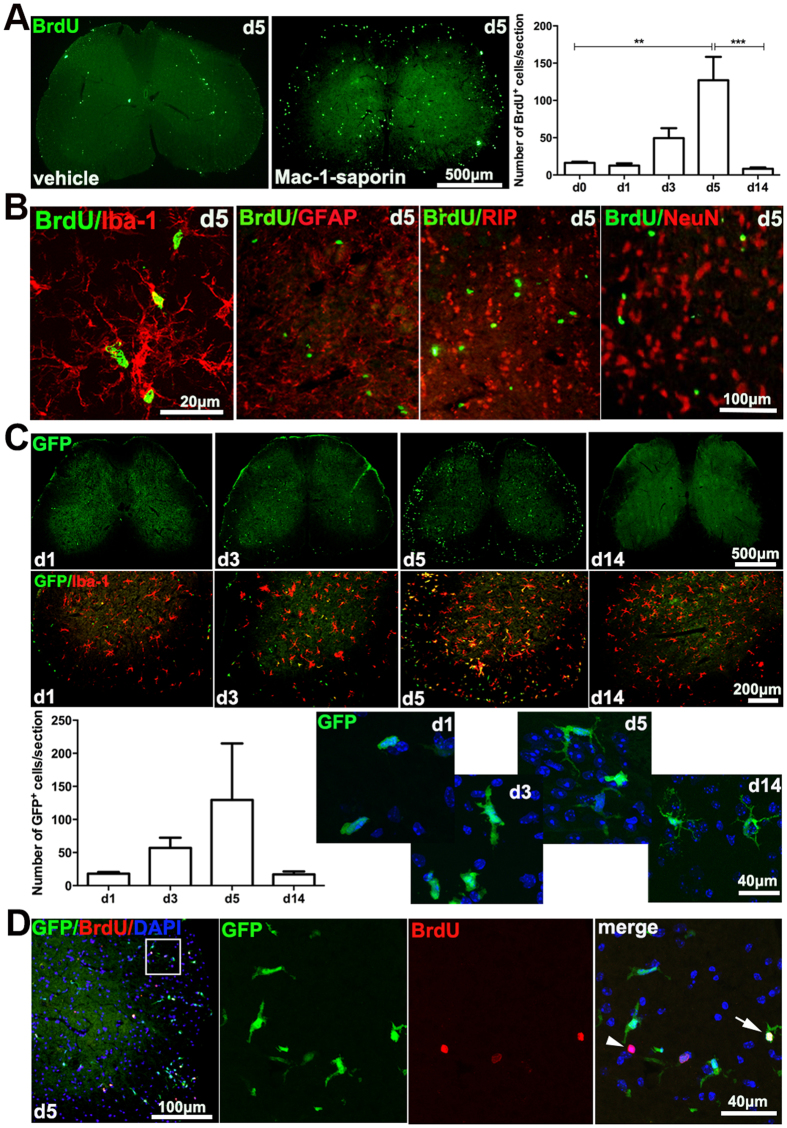
Microglia depletion-triggered cell proliferation and monocyte infiltration. (**A**) Following an intrathecal injection of Mac-1-saporin, there was a significant increase of BrdU^+^ cells in the lumbar spinal cord, which was peaked at day 5. N = 5 sections /mouse, 3–4 animals /group, *p < 0.05; **p < 0.01; ***p < 0.001. (**B**) Almost all BrdU^+^ cells were found co-localized with microglial cell marker, Iba-1. None of these proliferating cells were co-localized with GFAP, RIP, or NeuN, markers for astrocytes, oligodendrocytes and neurons, respectively. (**C**) GFP^+^ cells were found in the spinal parenchyma after Mac-1-saporin treatment in GFP chimeric mice, which was minimum at day 1, increased at day 3 and peaked at day 5, then almost no detectable at day 14. Infiltrated GFP^+^ cells exhibited different morphology at different time points, including round, elongated and ramified shapes. Note that not all GFP^+^ cells were Iba-1^+^, some round shaped GFP^+^ cells, detected at early time points did not express Iba-1, indicating these are newly infiltrated monocytes. N = 5 sections/mouse, 3 mice/group. (**D**) Some BrdU^+^ cells were co-localized with GFP (arrow), suggesting that infiltrated cells were also proliferating, while BrdU single labeled cells (arrowhead) were proliferating resident microglia.

Following an intrathecal injection of Mac-1-saporin, the number of BrdU^+^ cells significantly increased, peaked at day 5 (Fig. 2A). Almost all proliferating BrdU^+^ cells were Iba-1^+^ microglia, none of them were found colocalized with markers for other types of cells, such as GFAP (astrocytes), RIP (oligodendrocytes), or NeuN (neurons) (Fig. 2B). However, in GFP chimeric mice where rodent bone marrow-cells were replaced by GFP^+^ ones, a significant amount of GFP^+^ cells were detected in the lumbar spinal cords following Mac-1-saporin injection. Bone marrow derived monocyte infiltration was minimum at day 1, increased at day 3, peaked at day 5 and barely detectable at day 14 (Fig. 2C). At day 1–3, the infiltrated GFP^+^ cells were mostly in round or amoeboid shape. Gradually, these GFP^+^ cells became elongated and ramified with time passing on (day 5–14). Almost all ramified GFP^+^ cells found at day 5–14 were immunoreactive to Iba-1, indicating that these infiltrated monocytes had already differentiated into microglia. However, some round shaped GFP^+^ cells were single labeled, suggesting this subgroup of GFP^+^ cells were newly infiltrated monocytes which do not express or express low levels of Iba-1 (Fig. 2C). It is interesting to notice that both GFP-, resident microglia (Fig. 2D, arrowhead) and GFP^+^ infiltrated cells (Fig. 2D, arrow) could be co-localized with BrdU, thus both resident and monocyte-derived microglia proliferate following spinal microglia depletion. Infiltration of circulating monocytes into the spinal cord following microglia depletion was not only found in GFP chimeric mice, but also in normal C57BL6 mice using FACS analysis. A group of CD11b^+^CD45^high^ cells was detected in the spinal cord, 5 days post-Mac-1-saporin injection (Fig. 5C).

### Microglia depletion results in an acute local inflammatory reaction in the lumbar spinal cord

**Figure 3:**
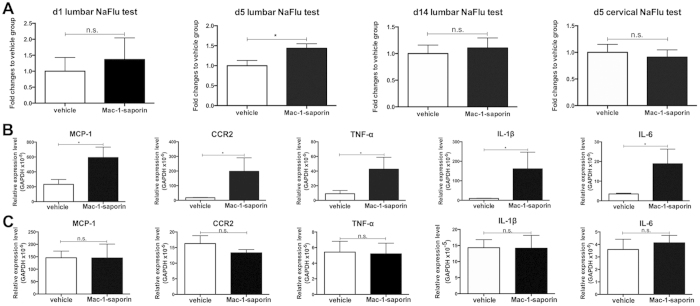
Evidence of acute inflammatory reaction in the spinal cord following Mac-1-saporin injection. Compared with vehicle treated animal, the amount of NaFlu in the lumbar spinal cord of Mac-1-saporin treated mice was increased, peaked at day 5, indicating that microglia depletion was associated with a breakdown of the BSCB, specific at the lumbar area where the Mac-1-saporin was injected (**A**). N = 3–4/group, *<0.05. Microglia depletion triggered an acute inflammation in the spinal cord. The mRNA expression of inflammatory molecules such as MCP-1, CCR2, TNF-α, IL-1β and IL-6 was sharply increased at day 1 post-depletion (**B)**. The significant difference was no longer detectable at day 5 (**C**). N = 4/group, *<0.05.

The fact that bone marrow derived monocytes infiltrated into the spinal cords following Mac-1-saporin injection led us to question the integrity of the blood spinal cord barrier (BSCB). We therefore injected intravenously a fluorescent tracer, sodium fluorescein (NaFlu) at day 1, day 5 and day 14 after Mac-1-saporin treatment. Indeed, to compare with the vehicle treated group, a significant increase of NaFlu was detected in the lumbar spinal cord at day 5, indicating that microglia depletion triggered a transient BSCB disruption. Such compromise was restricted only in the area where microglia depletion occurred as the increase of NaFlu was not observed in the cervical spinal cords (Fig. 3A). Furthermore, we examined the expression of various inflammatory molecules in the spinal cords where microglia were depleted. Quantitative PCR analysis revealed a significant increase on mRNA expression of MCP-1, CCR2, TNF-α, IL-1β and IL-6 at day 1 post-depletion (Fig. 3B), which was no longer noticeable at day 5 (Fig. 3C). We believe that these inflammatory mediators known to contribute to BSCB breakdown could be released by apoptotic dying microglia following Mac-1-saporin treatment.

**Figure 4:**
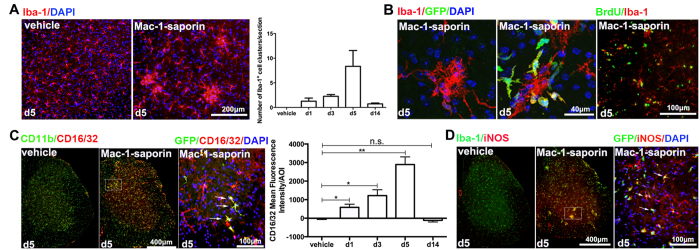
Presence of Iba-1^+^ cell clusters and M1-like microglia in the lumbar spinal cord during depletion-repopulation process. (**A**) Many Iba-1^+^ cell clusters were found in the spinal parenchyma following microglia depletion, which was peaked at day 5 post Mac-1-saporin treatment. N = 3–7 mice/group. (**B**) These microglia clusters were formed either solely by resident microglia or by the mixture of the resident and monocyte-derived microglia, where usually GFP^+^ cells were found outside, surrounding the clusters. Many Iba-1^+^ cells within the cell clusters were proliferating as they were colocalized with BrdU. (**C**) The expression of CD16/32 (Fcγ receptors III/II) was induced on microglia in mice received Mac-1-saporin treatment. Almost all CD16/32 ^+^ cells were co-localized with CD11b^+^ microglia, and all infiltrated GFP^+^ cells are immunoreactive to CD16/32. Quantitative analysis on the CD16/32 fluorescence intensity showed a progressive increase, peaking at day 5 and not detectable at d14. N = 5–7 sections/mouse, 3 mice/group, *p < 0.05; **p < 0.01. (**D**) iNOS expression was induced on microglia of mice received Mac-1-sapporin treatment, especially on microglia clusters. The majority of GFP^+^ cells expressed iNOS.

As depicted in Figs 1 and 4A, following depletion, some Iba-1^+^ cells aggregated together to form cell clusters, which appeared essentially during the acute phase, peaking at day 5. These clusters were made up either solely by resident microglia, or by the mixture of both resident and infiltrated microglia, where usually GFP^+^ cells were found outside, surrounding the clusters (Fig. 4B). Many cells within the clusters were BrdU^+^ (Fig. 4B), suggesting that newly generated microglia participated in the formation of clusters. To further determine functional characteristics of microglia during the depletion and repopulation process, we stained spinal microglia using well recognized M1 markers, e.g., CD16/32 (IgG receptors III and II, FcγR III/II) and iNOS. While almost non detectable on microglia of vehicle treated mice, CD16/32 expression was strongly induced on spinal microglia following Mac-1-saporin injection, which increased progressively, peaking again at day 5 (Fig. 4C), and fading away at day 14. Of note, virtually all CD16/32 positive signals were found on CD11b^+^ microglia and nearly all GFP^+^ infiltrated cells expressed CD16/32. Such M1-like pro-inflammatory microglia phenotype was further confirmed using the expression pattern of iNOS on CD11b^+^ and GFP^+^ cells (Fig. 4D).

### Cell depletion-triggered, MCP-1/CCR2 signaling-mediated transient inflammatory reaction is not required for the repopulation process

**Figure 5:**
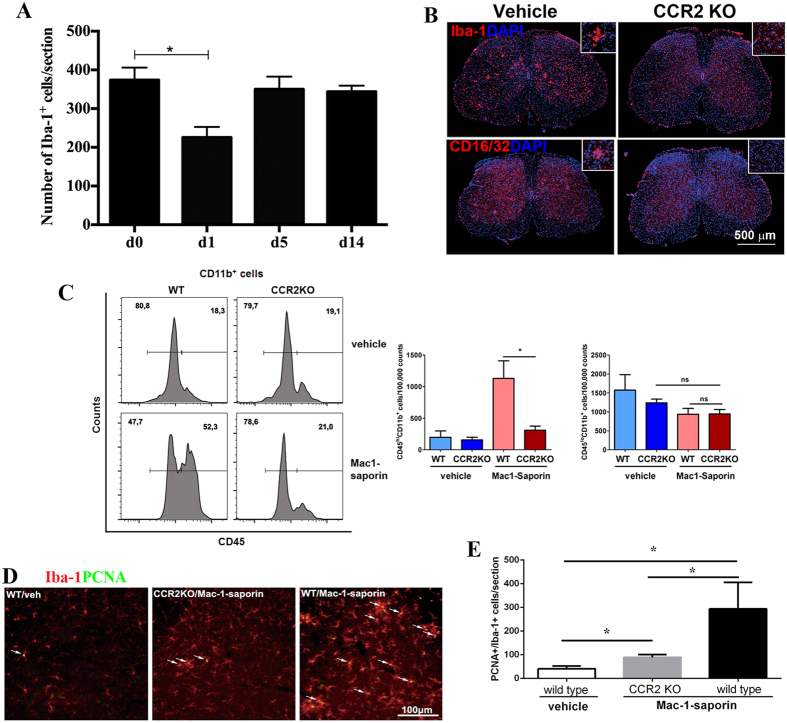
Microglia depletion and repopulation in CCR2 KO mice. (**A**) Following Mac-1-saporin depletion, the number of spinal cord microglia in CCR2KO mice dropped significantly at day 1 when compared with mice without depletion and returning to basal levels as soon as 5 days post-depletion. N = 5 sections/mouse, 3–4 mice/group, *p < 0.05; (**B**) Some characteristics seen in WT mice, e.g., presence of the cell clusters and robust expression of CD16/32 at the early stage of the repopulation (day 3–5) were absent or attenuated in CCR2KO mice. (**C**) FACS data showed that depletion-triggered recruitment of monocyte-derived microglia seen WT mice was almost completely prevented in CCR2 KO mice, since the CD11b^+^CD45^high^ subset in spinal cord of CCR2KO mice was significantly decreased, almost reached to the basal levels of either WT or CCR2KO mice without depletion. N = 4 mice/group, *p< 0.05; **p < 0.01. (**D**) Representative micrographs of PCNA expression in the spinal cords of WT mice treated with vehicle, CCR2KO mice and WT mice treated with Mac-1-saporin, at day 5 post-treatment. Almost all PCNA^+^ cells were colocalized with Iba-1^+^ cells (arrows). Quantitative analysis revealed that Mac-1-saporin triggered a significant increase of PCNA and Iba-1 double positive cells in CCR2KO mice; indicating that although local inflammation was largely attenuated, microglia in CCR2 KO mice were still able to proliferate following depletion. However, cell depletion triggered increase of PCNA^+^/Iba-1^+^ cell was 2 times higher in WT than in CCR2 KO mice. Such robust cell proliferation in WT mice was indeed part of inflammatory reaction, including proliferation of infiltrated circulating monocytes.

As MCP-1 expression increased shortly after depletion, and microglia migration (formation of cell clusters) and circulating monocyte infiltration were found during depletion-repopulation process, we were intrigued to examine the importance of MCP-1/CCR2 signaling in the course of microglia repopulation, since the requirement of this pathway in the recruitment if blood borne microglia has been well established in our previous study and in the literature^12^,^16^. To do this, we depleted microglia in CCR2 KO mice. Following a significant reduction of Iba-1^+^ cells at day 1 post-Mac-1-saporin injection, the total number of microglia cells were already recovered and stabilized to the basal level at day 5 (Fig. 5A). Interestingly, along with the process of repopulation, some characteristics of surviving and newly generated microglia observed in wild type (WT) mice, such as the presence of cell clusters and expression of CD16/32 at the early stage of the repopulation were absent in CCR2 KO mice (Fig. 5B). Furthermore, comparing with WT mice, 5 days post-depletion, CD11b^+^ CD45^high^ subset in the spinal cord of CCR2KO mice were significantly reduced, almost to the basal levels in either WT or CCR2 KO mice without depletion (Fig. 5C); indicating that depletion-triggered infiltration of circulating monocytes into the spinal parenchyma was prevented in mice deficient of CCR2. However, disrupting MCP-1/CCR2 signaling did not affect microglia self-renewal capability. Although much less than in WT Mac-1-saporin treated mice, the number of PCNA^+^/Iba-1^+^ cells in CCR2KO Mac-1-saporin treated mice was significantly higher than in WT vehicle treated mice (Fig. 5D). A large amount of PCNA^+^/Iba-1^+^ cells in WT Mac-1-saporin treated mice could indeed derive from inflammation associated cell proliferation, including the proliferation of infiltrating circulating monocytes (Fig. 2D).

### Spinal microglia responsiveness to peripheral nerve injury is not altered in newly generated population

**Figure 6:**
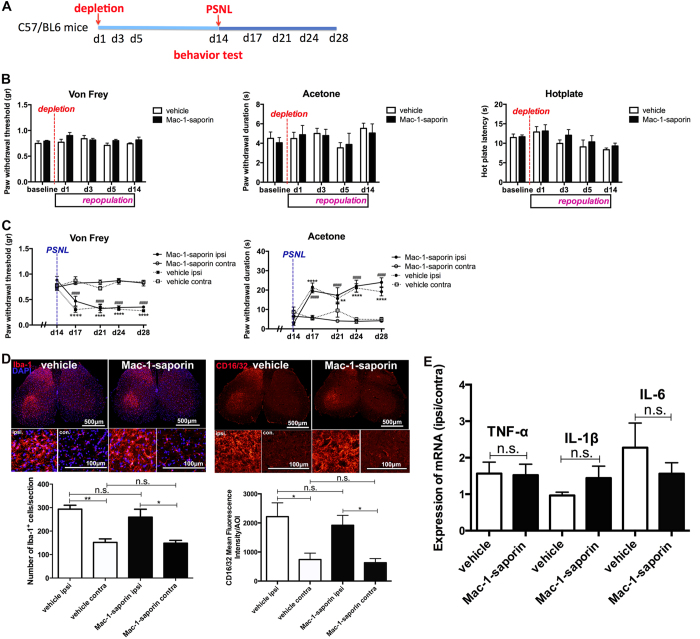
Impact of microglia depletion on normal pain behavior and response of newly generated microglia to peripheral nerve injury. (**A**) Experimental paradigm for monitoring pain behavior following microglia depletion and responsiveness of newly generated microglia to peripheral nerve injury. (**B**) Mechanical and thermal sensitivity was monitored for two weeks following microglia depletion. No significant differences were found between vehicle and Mac-1-saporin treated groups in von Frey, acetone and hot plate tests, indicating that microglia depletion and repopulation process did not affect normal pain behavior. N = 6 mice/group. (**C**) Mice with or without microglia depletion developed same pattern of neuropathic pain (mechanical and cold allodynia) following partial sciatic nerve ligation. N = 6 mice/group. (**D**) Fourteen days after cell depletion, when microglia repopulation was almost fully accomplished, original microglia (vehicle group) and newly generated microglia (Mac-1-saporin group) responded similarly to peripheral nerve injury. They generated same pattern of spinal microgliosis ipsilateral to the injury side, both expressed high levels of CD16/32. N = 5 sections/mouse, 3 mice/group, *p < 0.05; **p < 0.01. (**E**) There was no significant difference in the mRNA expression of TNF-α, IL-1β and IL-6 in lumbar spinal cords between vehicle- and Mac-1-saporin- treated groups. N = 3–6 mice/group.

It is well known that peripheral nerve injury can induce spinal microglial activation and activated microglia participate actively in the development and maintenance of injury triggered neuropathic pain. To examine the impact of spinal microglia depletion on both nociceptive and nerve injury triggered neuropathic pain behavior, and to understand the involvement of newly generated spinal microglia in these physiological and pathological processes, we first monitored animal pain behavior from day 1 to day 14 following spinal microglia depletion, then conducted partial sciatic nerve ligation (PSNL) at day 14 post-Mac-1-saporin injection where microglia repopulation is fully accomplished, pain behavior was assessed for two additional weeks (Fig. 6A). As demonstrated in Fig. 6B, shortly after the depletion and during the entire repopulation process, paw withdrawal thresholds in von Frey test, paw withdrawal duration in acetone test and withdrawal latency in hot plate test, were similar in both Mac-1-saporin and vehicle treated mice. This suggests that acute depletion of spinal microglia and the subsequent repopulation process did not alter animal mechanical and thermal sensitivity. The transient spinal inflammatory reaction following depletion (day1–day 5) was not sufficient to elicit neither allodynia nor hyperalgesia. The fact the microglia depletion did not alter mechanical and thermal sensitivity in naive mice further confirm that microglia involvement is not required in physiological nociceptive response. Furthermore, compared with vehicle treated animals, mice having newly generated spinal microglia developed similar pattern of mechanical and cold allodynia following PSNL injury (Fig. 6C). Same as original ones without depletion, newly generated spinal microglia in Mac-1-saporin treated mice responded to the insult of peripheral nerve injury. There was no significant difference between two groups, in terms of injury triggered spinal microgliosis and the increased expression of CD16/32 (Fig. 6D). The mRNA expression of TNF-α, IL-1β and IL-6 at 14 days post-PSNL was also similar in vehicle and Mac-1-saporin treated groups (Fig. 6E).

## Discussion

Microglia are tissue resident macrophages that occupy all regions of the central nervous system, including the brain, the spinal cord and the retina. Recent studies have shown that microglia not only act as sentinel cells to serve immune-related functions, they also have a number of key features in refining neuronal network^17^. It is thus necessary to achieve a true mechanistic understanding of how these cells are maintained in response to any internal and/or external insults. By focusing on spinal microglia, we demonstrated in this study that 1) following an acute depletion, microglia repopulation occurs quickly and its homeostasis can be re-established within 2 weeks; 2) self-renewal is the driving force in response to an abrupt microglia cell loss in the spinal cord; 3) acute microglia depletion, namely cell death, can trigger a transient inflammatory reaction that includes recruitment of monocyte-derived microglia, which is MCP-1/CCR2 signaling dependent, that is not required for microglia recovery; 4) newly generated microglia respond in the same manner as original ones to peripheral nerve injury; they contribute to the development of injury-associated neuropathic pain.

Physiological turnover, through which old differentiated cells are regularly eliminated and replaced by newly generated cells, is crucial in maintaining tissue homeostasis. Although microglia turnover in adulthood is slow^18^, cell loss in response to environmental stress, such as UV radiation, or accidental tissue damage, can be frequent in humans and has been replicated in various animal models. It is hence important to understand the endogenous monitoring system that ensures the repopulation in a proper way depending on their sensitivity to damage and/or their potential to proliferate and regenerate. In the tissue homeostasis system, in response to sudden, unexpected cell death, the primary strategy that cells use to compensate cell loss is to trigger divisions of the remaining cells, also called compensatory proliferation^19^. As observed in the current study and reported previously^5^,^6^, it is apparent that depletion of microglia results in proliferation of surviving microglia, which is indeed a highly coordinated and tightly controlled cellular response. Cell proliferation is initiated shortly after microglia ablation; however, it is strictly regulated where the equilibrium between cell death and cell division has been well maintained to prevent over-population. Bruttger *et al.*^6^ demonstrated that IL-1R signaling is required in the restorative proliferation process of microglia following depletion. Although thus far not fully defined in microglia repopulation, some other critical signaling molecules and pathways have been identified in *Drosophila* and mammals for their involvement of an apoptosis induced cell proliferation and differentiation^20^,^21^. For example, the caspase inhibitor p35 and JNK activation have been well documented for their key roles in preventing over-proliferation^22^. EGFR^23^ and Ptch1/Shh^24^ pathways were activated and involved in tumor cell repopulation. Another strategy to counteract the cell loss is through compensatory cellular hypertrophy (CCH), regulated probably by insulin/insulin-like growth factor (IGF) signaling pathway^25^. In our study, it appears that the CCH occurred earlier than cell proliferation. When the most prominent evidence of microglia hypertrophy was detected at day 1 post-Mac-1-saporin injection, the peak of cell proliferation was found at day 5, though, along with the increase of cell density, the CCH decreases. Probably guided by different signals and through different activation pathways, following local depletion, remaining spinal microglia carry out both CCH and compensatory proliferation to maintain the integrity of spinal micro-environment, which can be of major significance in regeneration processes for CNS tissue recovery; it might also have pathological relevance for CNS tumor growth.

The origins of microglial cells during physiological turnover and pathological recovery have been extensively debated. Microglia are of embryonic origin^26^,^27^. Monocytes found in the blood circulation are mononuclear leukocytes, constantly generated in the bone marrow from hematopoietic stem cells. It has been clearly demonstrated that circulating monocytes can infiltrate into the CNS parenchyma in pathological conditions. For instance, massive infiltration of monocytes has been found in demyelinating lesions in experimental autoimmune encephalomyelitis (EAE)^28^ and in peripheral nerve injury triggered spinal microglia activation^12^. These monocyte-derived microglia trigger EAE progression and contribute to the development of neuropathic pain respectively, but they vanish during the restoration of local homeostasis^12^,^28^. They do not permanently contribute to the resident microglia pool. Here we reveal that during a microglia ablation-triggered repopulation process, circulating monocytes actively populate the spinal cord parenchyma, giving rise to cells that are phenotypically indistinguishable from resident microglia, but these infiltrated cells remain in the spinal cords only transiently. They do not contribute to the resident microglia pool either. This might be a consequence of cell competition between infiltrated monocyte-derived microglia and CNS resident microglia^29^. Although both resident and monocyte-derived microglia proliferate following depletion, it seems that the infiltrated ones are the losers during competition. We speculate that they could indeed be eliminated by apoptosis and/or even get engulfed by neighboring winners, namely resident microglia. It is also possible that CNS environment does not favor circulating monocyte survival. They are at a growth disadvantage. All in all, it is not clear whether they undergo apoptosis or whether they exit the CNS compartment and migrate back to the circulation. Overall, in line with previous studies^5^,^6^, we confirm that resident microglia are able to maintain the stability of the cell population by relying on their self-renewal capability, independent of monocytes. The concept of potential cell competition between resident and monocyte-derived microglia is novel. Dissecting underlying molecular mechanisms of such cell competition could provide new approaches to elucidate distinct roles of resident and monocyte-derived microglia in many CNS pathophysiolocal conditions.

Infiltration of circulating monocytes into the CNS has been attributed to alterations in CNS environment, mainly local inflammation^6^,^30^. Mac-1-saporin leads to microglia cell death, which is associated with an acute inflammatory response in the spinal cord, including increased expression of proinflammatory cytokines/chemokines, and subsequent disruption of the BSCB. Several lines of evidence grounded the critical roles of MCP-1/CCR2 signaling in CNS monocyte recruitment during a variety of inflammatory, infective and traumatic conditions^31^,^32^,^33^. Our previous studies demonstrated that following peripheral nerve injury, MCP-1released by damaged neurons not only attract circulating monocyte to infiltrate into the spinal cord^12^, but also contribute to the breakdown of the BSCB^30^. Through the same mechanism by interacting with CCR2 on monocytes/microglia^34^ and on endothelial cells^35^, MCP-1 released by dying microglia promotes secondary spinal inflammation, including recruitment of monocytes, formation of microglia cell aggregates and induction of pro-inflammatory molecule expression on surviving spinal microglia. All these inflammation associated signals were significantly attenuated or no longer detectable in CCR2KO mice receiving the same amount of Mac-1-saporin treatment. Thus, our current microglia repopulation study confirmed again that recruitment of monocyte-derived microglia into the CNS is the consequence of local inflammation, dependent on MCP-1/CCR2 signaling. Remarkably, ruling out MCP-1-mediated secondary inflammation, including blocking recruitment of monocyte-derived microglia, did not affect depletion triggered-microglia repopulation. Microglia in CCR2KO mice are able to regain their stability in a similar timely manner as seen in WT mice, which presumably is ensured by the proliferation of resident microglia. This finding indicates that, acute, transient inflammatory reactions including recruitment of monocyte-derived microglia are just a separate response to cell depletion. This process is not required for the reset of microglia cell population to the steady status.

Previous studies reported that microglia depletion in adult mouse brain does not affect learning, memory and motor function^5^. Here we add up that spinal microglia depletion does not affect mouse sensory response, suggesting that some physiological functions might not need the involvement of microglia. Although recent study uncovered that male and female mice use different immune cells to mediate mechanical sensitivity^36^, the responsiveness of spinal microglia to peripheral nerve injury in male rodents and their contribution to the development of neuropathic pain have been well recognized in various animal models^37^. An injury to peripheral nerve can trigger activation of spinal microglia, which is associated with the release of numerous mediators, capable of sensitizing directly surrounding spinal neurons^38^. Removing microglia, inhibiting microglia activation or blocking microglia-to-neuron signaling, with either genetic or pharmacological approaches, all can attenuate injury triggered neuropathic pain behavior^39^,^40^. We showed that following depletion, microglia population regained its equilibrium in turns of cell numbers through cell proliferation. However, the functional status of these newly generated cells depend largely on the expression of functional receptors, release of cytokines/trophic factors, as well as their performance in response to various insults. Our results testify that newly generated microglia gain full capacity to react to the challenge of nerve injury. They behave as the original ones in induction of spinal microgliosis and secretion of inflammatory cytokines in the wake of an injury to peripheral nerve. Their participation in the genesis of neuropathic pain is proved by the fact that mice having either original microglia or newly repopulated microglia displayed identical pattern of injury triggered mechanical and cold allodynia.

To summarize, we confirm that similarly to microglia in the brain, spinal microglia can repopulate rapidly following elimination, which is driven essentially by a self-renewal process. Bone marrow-derived monocyte infiltration is part of an acute inflammatory reaction triggered by cell death. This infiltration is dependent on MCP-1/CCR2 signaling, but not required for re-establishment of microglia homeostasis. Newly generated microglia acquire functional characteristics. They are able to respond to nerve injury and participate in the generation of injury associated neuropathic pain.

## Methods

### Animals

Experiments were carried out in adult male C57BL/6 mice (20 to 25 g) (Charles River Laboratories, Quebec, Canada), green fluorescent protein (GFP) chimeric mice where rodent bone marrow-cells were replaced with GFP^+^ ones^41^ (CHUL Research Center, Laval University, Dr. S. Rivest) and CCR2KO mice (Jackson Lab, Bar Harbar, ME, USA). Mice were housed 4 to 5 per cage, in a temperature- and humidity- controlled vivarium, on a 12:12-hour light/dark cycle beginning at 7:00 am, with access to rodent chow and water ad libitum. Behavioral experiments were conducted between 8:00 am to 4:00 pm. All protocols were conducted according to the guidelines of the Canadian Council on Animal Care and the International Association for the Study of Pain, approved by the Institutional Animal Care and Use Committee of McGill University (Permit #5775).

### Spinal microglia depletion

To deplete microglia in the spinal cords, Mac-1-saporin (Advanced targeting system, San Diego, USA), a microglia selective toxin was injected intrathecally (7 μl/injection/mouse, 1.6 μg/μl) at the level of L4-L5. Vehicle groups received injection of same volume of either saline or saporin. No significant differences were observed in saline and saporin treated groups, data were pooled together.

### Nerve injury model

Partial sciatic nerve ligation (PSNL) was performed according to the method of Seltzer *et al.*^42^, adapted to mice^43^. Briefly, mice were anesthetized with isoflurane (3% for induction and maintenance), and under aseptic conditions the left sciatic nerve was exposed at high-thigh level. The dorsum of the nerve was carefully freed from surrounding connective tissues at a site near the trochanter just distal to the point at which the posterior biceps semitendinosus nerve branches off the common sciatic nerve. An 8-0 silk suture was inserted into the nerve with a 3/8 curved, reversed-cutting mini-needle, and tightly ligated so that the dorsal 1/3 to 1/2 of the nerve thickness was trapped in the ligature. The wound was then closed with 2 to 3 skin sutures (4-0).

### Pain behavior test

Mice were habituated to the testing environment daily for at least 2 days before baseline testing. The investigator was blinded to the treatments that the mice received.

Von Frey Test was performed to test paw sensitivity to mechanical stimuli. Calibrated monofilaments were applied to the plantar surface of the hindpaw and the 50% threshold to withdraw was calculated as previously described^12^. A decrease in threshold suggests the development of mechanical allodynia.

Acetone Test was used to evaluate sensitivity to cold stimuli. Total duration of acetone-evoked behaviors (flinching, licking or biting) was measured for 1 minute after one drop of acetone (~25 μl) was applied to the plantar surface of the hindpaw. An increase of withdrawal duration indicates the cold allodynia.

Hot plate (55 °C) was used as an unpleasant sensory heat stimulus to measure pain response. The latency to paw-licking, squeaking, or distressful behavior was measured. A decrease in latency suggests the development of heat hypersensitivity

### Immunohistochemistry (IHC)

Mice were deeply anesthetized with a ketamine/xylazine cocktail and then perfused transcardially with 0.9% saline followed by 4% paraformaldehyde in 0.1 M sodium phosphate buffer (pH 7.4). The lumbar spinal cords were removed and placed in the same fixative overnight, then transferred to 30% sucrose for cryoprotection. Frozen spinal cords were cut transversely into 25 μm-thick sections on a sliding microtome, collected in an anti-freeze solution [0.05 M sodium phosphate buffer (pH 7.3) containing 30% ethylene glycol and 20% glycerol] and stored at −20 °C until use. Standard fluorescent immunohistochemistry protocols were applied with the use of antibodies and dye against selective proteins. Floating sections were washed in TBS 3 times, blocked and incubated for 24 h at 4 °C with primary antibodies: rabbit anti-Iba-1 polyclonal antibody (ionizing calcium-binding adaptor molecule, for microglia and macrophages, 1:1000; Wako, Richmond, VA), CD16/32 (Fcγ receptors III/II) (1:400, R&D Systems, Minneapolis, MN), iNOS (1:200, BD Bioscience). Sections were then incubated for 60 min at room temperature with a corresponding secondary antibody, then counterstained with 4′, 6-diamidino-2-phenylindole (DAPI) for nuclear labeling. After rinses in TBS, sections were mounted onto slides and coverslipped with Vectashield Mounting medium (Vector Lab, Burlingame, CA).

### Cell proliferation assay

BrdU (50 mg/kg) was injected intraperitoneally at different time points (d1, d3, d5 and d14 post-i.t. Mac-1-saporin), 2 hours before the sacrifice. Cell proliferation was determined using incorporation of BrdU as index. To label cells incorporated with BrdU, free-floating sections were pretreated for 15 min in 1 N HCl at 37 °C, 20 min in 0.1 M borate buffer and three times rinses in Tris-buffered saline (TBS), pH 7.6, at room temperature. Non-specific labeling was blocked with TBS + 0.25% Triton X-100, 1% BSA and 3% normal goat serum for 1 h. Monoclonal goat anti-rat antibody against BrdU (1:250, Accurate Chemicals, Westbury, NY) was incubated with tissue sections for 48 h at 4 °C. After primary antibody incubation, sections were rinsed in TBS and incubated in Alexa 488-conjugated goat anti-rat IgG (1:250) in blocking buffer for 1 h. To identify the phenotype of newly born cells, double fluorescent-immunolabeling was performed. Sections were pretreated with 1 N HCl as described above, then incubated with BrdU antibody, together with one of the following antibodies at 4 °C for 48 h: rabbit anti-Iba-1 polyclonal antibody (ionizing calcium-binding adaptor molecule, for microglia and macrophages, 1:1000; Wako, Richmond, VA), mouse anti-neuron-specific nuclear protein (NeuN) monoclonal antibody (for neurons, 1:1000; Chemicon, Temecula, CA), rabbit anti-glial fibrillary acid protein (GFAP) polyclonal antibody (for astrocytes, 1:1000; Dako, Carpinteria, CA), RIP (for oligodendrocytes, 1:1000; Chemicon, Temecula, CA). Sections were then incubated for 60 min at room temperature with a corresponding secondary antibody. After rinses in TBS, sections were mounted onto slides and coverslipped with Vectashield Mounting medium (Vector Lab, Burlingame, CA).

Cell proliferation was also assessed using the expression of proliferating cell nuclear antigen (PCNA) as index. After acidic antigen retrieval (8 mins incubation in pH 6 citrate buffer, at 80 °C), lumbar spinal cord sections (day 5 post-Mac-1-saporin treatment) were immunolabelled simultaneously with mouse anti-PCNA monoclonal antibody (1:400; Dako, Carpinteria, CA) and rabbit anti-Iba-1 polyclonal antibody (1:1000).

### Image analysis

Images were captured using an Olympus BX51 microscope (Tokyo, Japan) equipped with a colour digital camera (Olympus DP71). Representative confocal microscopy images were acquired using an Olympus confocal laser-scanning biological microscope (Fluoview 1000). Images of 100× magnification were digitized with confocal Z-stacks set at 1 μm intervals. Z-stacks were displayed as overlays. Images were digitized with a constant exposure time and gain. The number of Iba-1^+^ cells and the intensity of CD16/32 expression were quantified by using the software Image Pro Plus 6 (Media Cybernetics, Rockville, MD). The number of infiltrated Iba-1^+^, GFP^+^, BrdU^+^ and PCNA+/Iba-1^+^ cells was counted manually across the whole section. The investigator was blinded to the treatments that the mice received.

### Evaluation of BSCB permeability

Blood spinal cord barrier (BSCB) integrity was assessed using a micromolecular tracer sodium fluorescein (NaFlu). The protocol was adopted and modified from our previous studies^30^. Briefly, NaFlu was administrated intravenously (10%; 2 ml/kg) and allowed to circulate for 30 min. Then, mice were transcardially perfused with cold saline for 10 min to remove intravascular NaFlu. The spinal meninges were removed before tissue homogenization. Muscles were used as positive controls. Following tissue homogenization, the concentration of NaFlu in supernatant was measured at excitation wavelength of 440 nm and emission wavelength of 525 nm using a spectrophotofluorometer. A standard curve of different amounts of NaFlu was drawn under identical conditions of the assay for calculating dye concentrations in the spinal cord. The contents of NaFlu in the spinal cords were normalized with the value in the muscles of the corresponding animals. The final data was presented as fold changes vs vehicle groups.

### Real-time PCR for inflammatory mediators

Gene expression of MCP-1, CCR-2, TNF-α, IL-1β, and IL-6 in the lumbar spinal cord was measured using real time qPCR. Total RNA of lumbar spinal cords from vehicle or Mac-1-saporin treated groups was extracted using Trizol (Invitrogen, Carlsbad, CA) according to the manufacturer’s instructions. Total RNA (1 μg) was used as template for reverse transcription. Real-time quantitative PCR reactions (in triplicate) were processed with a Rotor-Gene Q real-time PCR cycler (Qiagen) using SYBR Green Supermix from BIO-RAD. GAPDH was used as the internal control. The sequences of primers used in Real-Time PCR were listed in Table 1.

### Flow cytometry

Lumbar spinal cords of mice from each group were obtained after being perfused transcardially with 0.9% saline. Tissue samples were diced into small pieces and put into the DMEM containing collagenase (1.6mg/ml) and DNAase (250 units/ml) for digestion in 37 °C incubator for 30min. After filtration and wash, single cell suspensions were blocked in 2.42G blocking buffer at 4 °C. Samples were then stained with specific fluorochrome-conjugated antibody (rat anti-mouse CD11b PerCP, 1:50, BD pharmingen; rat anti-mouse CD45 APC, 1:50, eBioscience) for 25 min at 4 °C. Staining specificity was identified by omitting antibodies, and correlation of spectral overlap was done by using negative and positive compensation beads. Cellular events were acquired using BD canto FACs machine and data was analyzed using Flow Jo software. We use CD11b^+^/CD45^low^ and CD11b^+^/CD45^high^ subsets to distinguish resident microglia and monocyte-derived microglia, respectively.

### Statistic analysis

All data are presented as mean ± SEM. Pain behavior data was compared using Two-way ANOVA followed by multiple group comparisons between two groups among different time points. One-way ANOVA following a Turkey’s multiple comparison and t test was chosen for other data as it is appropriate. The criterion for statistical significance was P < 0.05.

## Additional Information

**How to cite this article**: Yao, Y. *et al.* Dynamics of spinal microglia repopulation following an acute depletion. *Sci. Rep.* **6**, 22839; doi: 10.1038/srep22839 (2016).

## Figures and Tables

**Table 1:** Sequences of primers used for real time qPCR experiments.

	Forward	Reverse
GAPDH	GTGAAGGTCGGTGTGAAC	AATCTCCACTTTGCCACTG
MCP-1	CTACTCATTCACCAGCAAGA	TCAGCACAGACCTCTCTC
CCR2	AGAAGAGGGCATTGGATT	CGTGGATGAACTGAGGTA
TNF-α	TTCTGTCTACTGAACTTC	CCATAGAACTGATGAGAG
IL-1β	CTATACCTGTCCTGTCTA	GCTCTTGACTTCTATCTTG
IL-6	CTGAAACTTCCAGAGATA	TTCATGTACTCCAGGTAG

## References

[b1] Ousman S. S. & Kubes P. Immune surveillance in the central nervous system. Nat Neurosci 15, 1096–1101 (2012). 22837040 10.1038/nn.3161 PMC7097282

[b2] Katsumoto A., Lu H., Miranda A. S. & Ransohoff R. M. Ontogeny and functions of central nervous system macrophages. J Immunol 193, 2615–2621 (2014). 25193935 10.4049/jimmunol.1400716 PMC4157312

[b3] Parkhurst C. N. *et al.* Microglia promote learning-dependent synapse formation through brain-derived neurotrophic factor. Cell 155, 1596–1609 (2013). 24360280 10.1016/j.cell.2013.11.030 PMC4033691

[b4] Wake H., Moorhouse A. J., Miyamoto A. & Nabekura J. Microglia: actively surveying and shaping neuronal circuit structure and function. Trends Neurosci 36, 209–217 (2013). 23260014 10.1016/j.tins.2012.11.007

[b5] Elmore M. R. *et al.* Colony-stimulating factor 1 receptor signaling is necessary for microglia viability, unmasking a microglia progenitor cell in the adult brain. Neuron 82, 380–397 (2014). 24742461 10.1016/j.neuron.2014.02.040 PMC4161285

[b6] Bruttger J. *et al.* Genetic Cell Ablation Reveals Clusters of Local Self-Renewing Microglia in the Mammalian Central Nervous System. Immunity 43, 92–106 (2015). 26163371 10.1016/j.immuni.2015.06.012

[b7] Varvel N. H. *et al.* Microglial repopulation model reveals a robust homeostatic process for replacing CNS myeloid cells. Proc Natl Acad Sci USA 109, 18150–18155 (2012). 23071306 10.1073/pnas.1210150109 PMC3497743

[b8] Hart A. D., Wyttenbach A., Perry V. H. & Teeling J. L. Age related changes in microglial phenotype vary between CNS regions: grey versus white matter differences. Brain Behav Immun 26, 754–765 (2012). 22155499 10.1016/j.bbi.2011.11.006 PMC3381227

[b9] Kettenmann H., Hanisch U. K., Noda M. & Verkhratsky A. Physiology of microglia. Physiol Rev 91, 461–553 (2011). 21527731 10.1152/physrev.00011.2010

[b10] Zhang J. & De Koninck Y. Spatial and temporal relationship between monocyte chemoattractant protein-1 expression and spinal glial activation following peripheral nerve injury. J Neurochem 97, 772–783 (2006). 16524371 10.1111/j.1471-4159.2006.03746.x

[b11] Echeverry S., Shi X. Q. & Zhang J. Characterization of cell proliferation in rat spinal cord following peripheral nerve injury and the relationship with neuropathic pain. Pain 135, 37–47 (2008). 17560721 10.1016/j.pain.2007.05.002

[b12] Zhang J. *et al.* Expression of CCR2 in both resident and bone marrow-derived microglia plays a critical role in neuropathic pain. J Neurosci 27, 12396–12406 (2007). 17989304 10.1523/JNEUROSCI.3016-07.2007 PMC6673247

[b13] Calvo M. & Bennett D. L. The mechanisms of microgliosis and pain following peripheral nerve injury. Exp Neurol 234, 271–282 (2012). 21893056 10.1016/j.expneurol.2011.08.018

[b14] Greenhalgh A. D. & David S. Differences in the phagocytic response of microglia and peripheral macrophages after spinal cord injury and its effects on cell death. J Neurosci 34, 6316–6322 (2014). 24790202 10.1523/JNEUROSCI.4912-13.2014 PMC6608120

[b15] Plemel J. R., Wee Yong V. & Stirling D. P. Immune modulatory therapies for spinal cord injury—past, present and future. Exp Neurol 258, 91–104 (2014). 25017890 10.1016/j.expneurol.2014.01.025

[b16] Dawson J., Miltz W., Mir A. K. & Wiessner C. Targeting monocyte chemoattractant protein-1 signalling in disease. Expert Opin Ther Targets 7, 35–48 (2003). 12556201 10.1517/14728222.7.1.35

[b17] Casano A. M. & Peri F. Microglia: multitasking specialists of the brain. Dev Cell 32, 469–477 (2015). 25710533 10.1016/j.devcel.2015.01.018

[b18] Lawson L. J., Perry V. H. & Gordon S. Turnover of resident microglia in the normal adult mouse brain. Neuroscience 48, 405–415 (1992). 1603325 10.1016/0306-4522(92)90500-2

[b19] Fan Y. & Bergmann A. Apoptosis-induced compensatory proliferation. The Cell is dead. Long live the Cell! Trends Cell Biol 18, 467–473 (2008). 18774295 10.1016/j.tcb.2008.08.001 PMC2705980

[b20] Poss K. D. Advances in understanding tissue regenerative capacity and mechanisms in animals. Nat Rev Genet 11, 710–722 (2010). 20838411 10.1038/nrg2879 PMC3069856

[b21] Gauron C. *et al.* Sustained production of ROS triggers compensatory proliferation and is required for regeneration to proceed. Sci Rep 3, 2084 (2013). 23803955 10.1038/srep02084 PMC3694286

[b22] Tamori Y. & Deng W. M. Compensatory cellular hypertrophy: the other strategy for tissue homeostasis. Trends Cell Biol 24, 230–237 (2014). 24239163 10.1016/j.tcb.2013.10.005 PMC4022146

[b23] Zips D. *et al.* Epidermal growth factor receptor inhibitors for radiotherapy: biological rationale and preclinical results. J Pharm Pharmacol 60, 1019–1028 (2008). 18644194 10.1211/jpp.60.8.0008

[b24] Chaudary N. *et al.* Hedgehog pathway signaling in cervical carcinoma and outcome after chemoradiation. Cancer 118, 3105–3115 (2012). 22028038 10.1002/cncr.26635

[b25] Tamori Y. & Deng W. M. Tissue repair through cell competition and compensatory cellular hypertrophy in postmitotic epithelia. Dev Cell 25, 350–363 (2013). 23685249 10.1016/j.devcel.2013.04.013 PMC3891806

[b26] Alliot F., Godin I. & Pessac B. Microglia derive from progenitors, originating from the yolk sac, and which proliferate in the brain. Brain Res Dev Brain Res 117, 145–152 (1999). 10567732 10.1016/s0165-3806(99)00113-3

[b27] Ginhoux F. *et al.* Fate mapping analysis reveals that adult microglia derive from primitive macrophages. Science 330, 841–845 (2010). 20966214 10.1126/science.1194637 PMC3719181

[b28] Ajami B., Bennett J. L., Krieger C., McNagny K. M. & Rossi F. M. Infiltrating monocytes trigger EAE progression, but do not contribute to the resident microglia pool. Nat Neurosci 14, 1142–1149 (2011). 21804537 10.1038/nn.2887

[b29] Tamori Y. & Deng W. M. Cell competition and its implications for development and cancer. J Genet Genomics 38, 483–495 (2011). 22035869 10.1016/j.jgg.2011.09.006 PMC3891807

[b30] Echeverry S., Shi X. Q., Rivest S. & Zhang J. Peripheral nerve injury alters blood-spinal cord barrier functional and molecular integrity through a selective inflammatory pathway. J Neurosci 31, 10819–10828 (2011). 21795534 10.1523/JNEUROSCI.1642-11.2011 PMC6623085

[b31] Huang D. R., Wang J., Kivisakk P., Rollins B. J. & Ransohoff R. M. Absence of monocyte chemoattractant protein 1 in mice leads to decreased local macrophage recruitment and antigen-specific T helper cell type 1 immune response in experimental autoimmune encephalomyelitis. J Exp Med 193, 713–726 (2001). 11257138 10.1084/jem.193.6.713 PMC2193420

[b32] Kelder W., McArthur J. C., Nance-Sproson T., McClernon D. & Griffin D. E. Beta-chemokines MCP-1 and RANTES are selectively increased in cerebrospinal fluid of patients with human immunodeficiency virus-associated dementia. Ann Neurol 44, 831–835 (1998). 9818943 10.1002/ana.410440521

[b33] Rancan M. *et al.* Upregulation of ICAM-1 and MCP-1 but not of MIP-2 and sensorimotor deficit in response to traumatic axonal injury in rats. J Neurosci Res 63, 438–446 (2001). 11223919 10.1002/1097-4547(20010301)63:5<438::AID-JNR1039>3.0.CO;2-P

[b34] Dal-Secco D. *et al.* A dynamic spectrum of monocytes arising from the *in situ* reprogramming of CCR2+ monocytes at a site of sterile injury. J Exp Med 212, 447–456 (2015). 25800956 10.1084/jem.20141539 PMC4387291

[b35] Dzenko K. A., Song L., Ge S., Kuziel W. A. & Pachter J. S. CCR2 expression by brain microvascular endothelial cells is critical for macrophage transendothelial migration in response to CCL2. Microvasc Res 70, 53–64 (2005). 15927208 10.1016/j.mvr.2005.04.005

[b36] Sorge R. E. *et al.* Different immune cells mediate mechanical pain hypersensitivity in male and female mice. Nat Neurosci 18, 1081–1083 (2015). 26120961 10.1038/nn.4053 PMC4772157

[b37] Grace P. M., Hutchinson M. R., Maier S. F. & Watkins L. R. Pathological pain and the neuroimmune interface. Nat Rev Immunol 14, 217–231 (2014). 24577438 10.1038/nri3621 PMC5525062

[b38] Ji R. R., Xu Z. Z. & Gao Y. J. Emerging targets in neuroinflammation-driven chronic pain. Nat Rev Drug Discov 13, 533–548 (2014). 24948120 10.1038/nrd4334 PMC4228377

[b39] Tsuda M., Masuda T., Tozaki-Saitoh H. & Inoue K. Microglial regulation of neuropathic pain. J Pharmacol Sci 121, 89–94 (2013). 23337437 10.1254/jphs.12r14cp

[b40] Zhang J. *et al.* Can modulating inflammatory response be a good strategy to treat neuropathic pain? Curr Pharm Des 21, 831–839 (2015). 25345609 10.2174/1381612820666141027115508

[b41] Turrin N. P., Plante M. M., Lessard M. & Rivest S. Irradiation does not compromise or exacerbate the innate immune response in the brains of mice that were transplanted with bone marrow stem cells. Stem Cells 25, 3165–3172 (2007). 17761757 10.1634/stemcells.2007-0508

[b42] Seltzer Z., Dubner R. & Shir Y. A novel behavioral model of neuropathic pain disorders produced in rats by partial sciatic nerve injury. Pain 43, 205–218 (1990). 1982347 10.1016/0304-3959(90)91074-S

[b43] Malmberg A. B. & Basbaum A. I. Partial sciatic nerve injury in the mouse as a model of neuropathic pain: behavioral and neuroanatomical correlates. Pain 76, 215–222 (1998). 9696476 10.1016/s0304-3959(98)00045-1

